# Mental disorders and criminal re-referrals in juveniles who sexually offended

**DOI:** 10.1186/s13034-015-0035-x

**Published:** 2015-02-25

**Authors:** Lisette A ‘t Hart-Kerkhoffs, Cyril Boonmann, Theo AH Doreleijers, Lucres MC Jansen, Anton Ph. van Wijk, Robert RJM Vermeiren

**Affiliations:** Department of Child and Adolescent Psychiatry and the EMGO Institute for Health and Care Research, VU University Medical Center Amsterdam, P.O. Box 303, 1115 ZG Duivendrecht, Amsterdam, The Netherlands; Bureau Beke Research and Consultancy, Arnhem, The Netherlands; Curium-LUMC, Leiden University Medical Center, Leiden, The Netherlands

**Keywords:** Sexual offending juveniles, Psychopathology, Mental disorders

## Abstract

**Objective:**

To investigate the prevalence of mental disorders in (subgroups of) juvenile suspects who sexually offended (JSOs), and its relation with criminal re-referrals five to eight years later.

**Methods:**

A sample of 106 JSOs (mean age 15.0 ± 1.5 years) referred to the Dutch Child Protection Board between May 2003 and December 2006 was classified into JSOs with child victims (N = 19), solo JSOs with adolescents and/or adults victims (N = 29), and group JSOs with adolescents and/or adults victims (N = 58). Mental disorders were assessed at baseline by means of a semi-structured interview (K-SADS-PL), the level of functioning by means of the Children’s Global Assessment Scale (CGAS) and re-referrals was ascertained from official judicial registration systems.

**Results:**

Three quarters of JSOs met criteria for at least one mental disorder. Comorbidity was found in more than half of the subjects and almost two thirds of the JSOs were functionally impaired. As compared to the other subgroups, JSOs with child victims showed higher rates of affective disorders and had a lower overall level of functioning. Furthermore, JSOs re-referred for another sexual offense were more often diagnosed with an affective disorder, were more often sexually abused and had a lower level of global functioning than JSOs who were not re-referred for another sex offense.

**Conclusions:**

JSOs should receive mental assistance, as more than two thirds are functionally impaired due to mental problems. This may not only be important to safeguard the development of the juvenile offender but might also reduce repeated sexual offending.

## Introduction

Sexual offending behavior of juveniles is often a reason for public concern. It is estimated that minors commit about 20% of all rapes and 30-50% of all child molestations [1]. A recent nationwide U.S. study of sex offender police records, demonstrated that 26% of all sex offenders in general and 36% of all sex offenders with juvenile victims were minors [2]. Although several studies have reported on the association of mental disorders and juvenile delinquency [3-5], only a limited number of studies have addressed the prevalence of mental disorders in juveniles who sexually offended (JSOs) (e.g., [6,7]). Even though these studies reported high prevalence rates of internalizing disorders as well as externalizing disorders, they did not allow for firm conclusions because of methodological shortcomings (e.g., limited number of JSOs, variety of sexual offenses). Furthermore, although previous research in juvenile offender populations found a positive relationship between mental disorders, especially externalizing disorders, and reoffending [8-10], the Galli et al. [6] and Kavoussi et al. [7] studies did not address recidivism. Examining mental disorders in JSOs in relation to reoffending may contribute to the issue to what extent psychiatric care should be offered to this specific subgroup.

Furthermore, JSOs constitute a heterogeneous group. Previous research reported differences in mental health problems between subgroups of JSOs. While in rapists, for example, externalizing problems are more common [11], offenders with a preference for (prepubescent) child victims are more likely to show internalizing problems [11,12], and were more often sexually abused themselves [13]. In addition, based on a retrospective analysis of justice files, Bijleveld and Hendriks [14] reported that group sex offenders had lower scores on the personality characteristics neuroticism, impulsivity and sensation seeking and higher scores on sociability as compared to solo sex offenders. Additionally, the subgroup of JSOs victimizing children in particular showed poorly developed social skills [15-18]. Although there has been much research on the mental health problems in various subgroups of JSOs, research on mental disorders is limited. Therefore, it may carry relevance to compare mental disorders in subgroups of JSOs, especially in subgroups based on age of victim preference [19,20], as well as on the number of offenders taking part in the sex offense (solo or group offenders) [17].

Sexual recidivism rates in JSOs were repeatedly found to be relatively low. A recent review and meta-analysis showed that only 7% of JSOs reoffended sexually [21]. This was in line with a review of Fortune and Lambie [22] reporting on average 10% reoffending in JSOs (ranging from 0% to 42%). However, the same studies revealed that JSOs are generally more likely to reoffend non-sexually (e.g., burglary, theft, robbery). Caldwell [21], for example, found a general re-offense rate of 43%. Although mental disorders have shown to be related to juvenile reoffending in general [8-10], the aforementioned studies regarding JSOs did not address the relationship between mental disorders and sexual reoffending. As it has been shown that juvenile offenders in mental treatment diversion were significantly less often rearrested within one year than juvenile offenders of a full waitlist comparison group [23], treating mental disorders is hypothesized to reduce re-referrals.

The overall aim of this study was to examine the prevalence of mental disorders in JSOs and to assess its relationship with repeated sexual offending behavior. More specifically, the prevalence of mental disorders will be studied in the group of JSOs, as well as in specific subgroups. In line with previous research, a distinction will be made between JSOs with child victims, solo JSOs with adolescent/adult victims, and group JSOs with adolescent/adult victims [18,24,25]. It is hypothesized that, whereas JSOs with child victims will demonstrate the most internalizing disorders, group JSOs with adolescent/adult victims will show the most externalizing disorders. In addition, the relation between mental disorders and re-referrals in JSOs will be examined. This knowledge will inform us to what extent psychiatric care should be given to (subgroups of) JSOs in order to reduce (re-referrals for) sexual offending behavior.

## Methods

### Participants

Participants in this prospective longitudinal study were 106 male juvenile suspects (aged 12 to 18) of a sexual offense referred to the Child Protection Board (CPB) between May 2003 and December 2006. For the sake of readability, these juvenile suspects will be referred to as juvenile(s) (suspects) who sexually offended (JSOs). Due to the limited number of female JSOs, all girls were excluded from the sample. As the police in the Netherlands is obliged to refer all 12 to 18 year old suspects of a sex crime to the CPB, regional CPB offices were the primary sites of inclusion. Out of twenty-two regional offices, four were selected because of their location spread over rural and urban regions. Also JSOs who immediately were admitted to four (out of the thirteen) pretrial Juvenile Justice Institutions in The Netherlands were asked to participate as well.

Exclusion criteria were an IQ below 70 and insufficient command of the Dutch language. The Ethics Committee of the VU University Medical Center Amsterdam, The Netherlands, approved the study. After explaining the study to the participants, we obtained written informed consent from them and their parents or legal representatives.

In total, 161 boys were eligible for participation, of which 106 agreed to participate (response rate = 66%) (Mean age: 15.0; SD: 1.5). Non-responders did not differ from responders with respect to age (F = 1.2; p = 0.28) or offense characteristics, such as gender of victim (*χ*^2^ = 0.00; p = 1.00), age of victim (*χ*^2^ = 0.84; p = 0.36) or type of offending (e.g., child abuse, group sex offense) (*χ*^2^ = 0.53; p = 0.77). Responders were more often of non-Dutch ethnicity than non-responders (76% versus 43%, *χ*^2^ = 14.30; p < 0.00).

Based on characteristics of the index referral (the sexual offense for which the juvenile was included in the study) (i.e., age of the victim and number of offenders) retrieved from both official registration systems and CPB files, JSOs were classified into three subgroups: a) JSOs with child victims, suspected of having sexually abused children (below 12 years of age) who were at least four years younger than the offender himself (N = 19); b) solo JSOs with adolescent/adult victims, suspected of having raped or sexually assaulted adolescents (at least twelve years old) or adults (N = 29); c) group JSOs with adolescent/adult victims, suspected of having raped or sexually assaulted adolescents (at least twelve years old) or adults (N = 58). The three subgroups were mutually exclusive.

### Instruments

#### K-SADS-PL

Mental disorders were assessed by means of the K-SADS-PL (Schedule for Affective Disorders and Schizophrenia for school age children - Present and Lifetime [26-28]), a semi-structured interview for mental disorders listed in the Diagnostic and Statistical Manual of Mental Disorders (DSM-IV [29]). Research has shown good concurrent validity and high interrater agreement. Test-retest reliability kappa coefficients have been described as good for present diagnoses of posttraumatic stress disorder (PTSD) and attention-deficit hyperactivity disorder (ADHD) and was excellent for present and/or lifetime diagnoses of major depression, any bipolar disorder, generalized anxiety disorders, conduct disorder (CD) and oppositional defiant disorder (ODD) [26,30]. Histories of sexual abuse and parental physical abuse were also investigated by means of the K-SADS-PL. Because of our interest in mental disorders prior to the index referral, the time frames of the K-SADS-PL were adapted from ‘last year’ into ‘year before arrest’ and ‘last six months’ into ‘last six months before arrest’. The K-SADS was generally assessed within one month after the arrest. The first author performed all K-SADS-PL interviews. She received formal training to carry out these interviews.

#### The Children’s Global Assessment Scale (CGAS)

The CGAS [31] provides a global measurement of the level of functioning in children and adolescents and was scored as part of the K-SADS-PL interview. A score below 61 indicates functional impairment and, therefore, a serious need for treatment.

#### Prior referrals and re-referrals

By means of the official judicial registration systems of the Dutch Ministry of Security and Justice (JDS: Justice Documentation System [Justitieel Documentatie Systeem]) both the referrals prior to the index referral and the re-referrals in the five to eight year follow-up period after the index referral were examined. Prior referrals were defined as referrals registered before the index referral. Re-referrals were defined as referrals registered after the index referral. With respect to offending behavior, a distinction was made between referrals for sexual offenses and non-sexual offenses. Sexual offenses were defined as sexual offenses according to the Dutch Criminal Code. Non-sexual offenses were all offenses according to the Dutch Criminal Code without sexual offenses. In the JDS records, violations that yield a more serious punishment when repeated were also included.

### Data analyses

The data were processed and analyzed with IBM SPSS 19 (International Business Machines corporation Statistical Package for the Social Sciences, version 19). For all calculations the level of statistical significance was set at .05. First, in order to determine the prevalence of mental disorders, the global assessment of functioning, and the prior referrals and re-referrals of the subjects, descriptive statistics were performed. Second, differences between subgroups of JSO ([a] JSOs with child victims, solo JSOs with adolescent/adult victims, and group JSOs with adolescent/adult victims, [b] JSOs re-referred for another sexual offense, JSOs re-referred for a non-sexual offense and JSOs who were not re-referred) were analyzed using Chi-square tests (or Fisher Exact when expected cell counts less than 5) for categorical variables, and ANOVA’s (analysis of variance, with Bonferroni correction for multiple comparisons) for continuous variables. It should be noted that using corrections as Bonferroni increases the risk for Type I errors.

## Results

### Prevalence rates of mental disorders and level of functioning

Prevalence rates of mental disorders and scores on the CGAS are shown in Table 1. Three quarters of the juvenile(s) (suspects) who sexually offended (JSOs) met criteria for at least one mental disorder, with comorbidity (more than one disorder in the same person) being present in more than half of the total group. Although a history of sexual abuse was reported by twelve percent and a history of parental physical abuse in more than one third, none of the participants met criteria for PTSD. The mean CGAS score for the group was 60 (SD = 12), with almost 70% having a score below 61, the recommended cut-off for psychiatric treatment.

**Table 1:** **Prevalence of mental disorders, level of functioning and history of abuse**

	**Total (N = 106)**	**JSOs with child victims (N = 19)**	**Solo JSOs (N = 29)**	**Group JSOs (N = 58)**		**Post Hoc**	
	**% (N)**	**% (N)**	**% (N)**	**% (N)**	***χ*** **2; df = 2; p**		**OR (95% CI)**
Any disorder	75 (79)	84 (16)	83 (24)	67 (39)	3.60; NS		
Comorbidity	54 (57)	74 (14)	69 (20)	40 (23)	10.37;*	b	4.3 (1.4-13.4)
						c	3.4 (1.3-8.7)
Any internalizing disorder	39 (41)	63 (12)	38 (11)	31 (18)	6.23;*	b	3.8 (1.3-11.3)
- Any anxiety disorder	33 (35)	47 (9)	30 (8)	30 (18)	2.26; NS		
- Any affective disorder	12 (13)	42 (8)	14 (4)	2 (1)	21.78;*	a	4.5 (1.1-18.3)
						b	41.5 (4.7-365.5)
						c	9.1 (1.0-85.8)
DBD	57 (60)	53 (10)	69 (20)	52 (30)	2.48; NS		
- CD	40 (42)	37 (7)	55 (16)	33 (19)	4.14; NS		
- ODD	46 (49)	53 (10)	52 (15)	41 (24)	1.21; NS		
ADHD	31 (33)	58 (11)	42 (12)	17 (10)	12.99;*	b	6.6 (2.1-20.6)
						c	3.3 (1.2-9.3)
SUD	12 (13)	21 (4)	22 (6)	5 (3)	5.98;*	b	4.9 (1.0-24.3)
						c	4.8 (1.1-20.8)
Parental physical abuse	37 (39)	42 (8)	41 (12)	33 (19)	0.90; NS		
History of sexual abuse	12 (13)	47 (9)	3 (1)	5 (3)	26.57;*	a	25.2 (2.8-224.8)
						b	16.5 (3.8-71.8)
CGAS < 61	64 (68)	84 (16)	72 (21)	53 (31)	7.07;*	b	4.6 (1.2-17.7)
Mean CGAS (SD)	60 (12)	51 (12)	59 (12)	63 (11)	F_(2, 103)_: 7.84;*	a,b	

*Significant difference at the .05 level, NS = not significant.

a significant difference between JSOs with child victims and solo JSOs; b significant difference between JSOs with child victims and group JSOs; c significant difference between solo and group JSOs.

DBD = Disruptive Behavior Disorder; CD = Conduct Disorder; ODD = Oppositional Defiant Disorder; ADHD = attention-deficit hyperactivity disorder; SUD = Substance Use Disorder; CGAS = Children’s Global Assessment Scale; SD = Standard Deviation; N = Number; df = degrees of freedom; OR = Odds Ratio; CI = Confidence Interval.

### Comparison of subgroups of JSOs

Although subgroups did not differ in presence of any mental disorder, comorbidity was least present in the group JSOs with adolescent/adult victims (see Table 1).

As for specific mental disorders, JSOs with child victims showed higher rates of internalizing disorders, affective disorders, and ADHD than group JSOs. They also had a history of sexual abuse more often than group JSOs. Compared to solo JSOs with adolescent/adult victims, JSOs with child victims showed a higher prevalence of affective disorders and reported sexual abuse more often. Finally, solo JSOs had higher rates of comorbidity and a higher prevalence of affective disorders, and ADHD, than group JSOs.

As for global functioning, JSOs with child victims were characterized by an overall lower CGAS score compared to both the solo and group JSOs with adolescent/adult victims and by a larger number of individuals scoring <61 than in the subgroup of group offenders.

### Prior referrals and re-referrals

With regard to official judicial registration records, information of 104 JSOs (out of 106 JSOs) was retrieved. The index referral was found in 98 cases. In 8 cases we could not retrieve information about the juvenile offender and/or the index referral from the official judicial registration system. Of the 98 JSOs, 41% were referred for a sexual offense preceding the index referral (Table 2). Whereas 7% was re-referred for another sexual offense, 80% was re-referred for a non-sexual offense. Subgroups did not significantly differ in prior referrals or re-referrals.

**Table 2:** **Prior referrals and re-referrals**

	**Total (N = 98)**	**JSOs with child victims (N = 18)**	**Solo JSOs (N = 28)**	**Group JSOs (N = 52)**	
	**% (N)**	**% (N)**	**% (N)**	**% (N)**	***χ*** **2; df = 2; p**
Prior to index referral					
Total offense history	64 (63)	61 (11)	68 (19)	64 (33)	0.25; .NS
- 1 > sexual offense	41 (40)	50 (9)	46 (13)	35 (18)	1.82; .NS
- Other offense	54 (53)	39 (7)	50 (14)	62 (32)	3.02; .NS
Re-referral					
Total reoffending	82 (80)	78 (14)	71 (20)	89 (46)	3.74; .NS
- Sexual reoffending	7 (7)	11 (2)	7 (2)	6 (3)	0.58; .NS
- Other reoffending	80 (78)	67 (12)	71 (20)	89 (46)	5.52; .NS

NS = not significant.

N = Number; df = degrees of freedom.

JSOs re-referred for another sexual offense were diagnosed more often with an affective disorder than both JSOs re-referred for a non-sexual offense and JSOs who were not re-referred at all. JSOs re-referred for another sexual offense were also more often sexually abused than JSOs re-referred for a non-sexual offense, and had a significantly lower level of global functioning than JSOs who were not re-referred at all (Table 3).

**Table 3:** **Prevalence of mental disorders, level of functioning and history of abuse in JSOs re-referred for another sexual offense, re-referred for another non-sexual offense and not re-referred at all**

	**Sexual re-referral (N = 7)**	**General re-referral (N = 73)**	**Non re-referral (N = 18)**		**Post Hoc**	
	**% (N)**	**% (N)**	**% (N)**	***χ*** **2; df = 2; p**		**OR (95% CI)**
Any disorder	100 (7)	77 (56)	61 (11)	4.35; .11		
Comorbidity	71 (5)	56 (41)	44 (8)	1.61; .45		
Any internalizing disorder	71 (5)	36 (26)	39 (7)	3.45; .18		
- Any anxiety disorder	43 (3)	30 (22)	39 (7)	0.86; .65		
- Any affective disorder	57 (4)	10 (7)	11 (2)	12.64;*	a	12.6 (2.3-63.1)
					b	10.7 (1.3-86.9)
DBD	57 (4)	62 (45)	39 (7)	3.05; .22		
- CD	29 (2)	48 (35)	17 (3)	6.32;*	c	4.6 (1.2-17.3)
- ODD	57 (4)	48 (35)	33 (6)	1.62; .44		
ADHD	43 (3)	30 (22)	33 (6)	0.51; .78		
SUD	29 (2)	12 (9)	11 (2)	1.56; .46		
Parental physical abuse	14 (1)	40 (29)	33 (6)	1.89; .39		
History of sexual abuse	43 (3)	8 (6)	22 (4)	8.20;*	a	8.4 (1.5-46.5)
CGAS < 61	100 (7)	66 (48)	50 (9)	5.59; .06		
Mean CGAS (SD)	49 (9)	60 (11)	63 (13)	F_(2, 95)_: 4.03;*	b	

*Significant difference at the .05 level, NS = not significant.

a significant difference between JSOs with a sexual re-referral and JSOs with a general re-referral; b significant difference between JSOs with a sexual re-referral and JSOs without a re-referral; c significant difference between JSOs with a general re-referral and JSOs without a re-referral.

DBD = Disruptive Behavior Disorder; CD = Conduct Disorder; ODD = Oppositional Defiant Disorder; ADHD = attention-deficit hyperactivity disorder; SUD = Substance Use Disorder; CGAS = Children’s Global Assessment Scale; SD = Standard Deviation; N = Number; df = degrees of freedom; OR = Odds Ratio; CI = Confidence Interval.

## Discussion

In the current study, we examined mental disorders in juvenile suspects of a sexual offense, as well as subgroups of these suspects. For the sake of readability, these juvenile suspects will be referred to as juvenile(s) (suspects) who sexually offended (JSOs). Furthermore we examined differences in mental disorders between JSOs re-referred for another sexual offense, re-referred for a non-sexual offense, and without a re-referral. In total, over three-quarters were found to meet criteria for at least one mental disorder, with over half being diagnosed with comorbid conditions, and two thirds being functionally impaired. Rates differed between subgroups of JSOs, with JSOs with child victims showing the most mental disorders. With regard to re-referrals, 7% of JSOs was re-referred for another sexual offense, whereas 80% was re-referred for a non-sexual offense. JSOs re-referred for a sexual offense were more often diagnosed with an affective disorder, were sexually abused more often and had a lower level of global functioning than JSOs who were not. This study indicates that the mental health needs of JSOs deserve special attention.

The current study suggests that JSOs have relatively low prevalence rates of CD and SUD and high rates of ADHD and internalizing disorders as compared to findings on general offenders [3-5]. First, the relatively low prevalence of CD and SUD indicates that not all sex offenses may be committed in the context of a problematic antisocial behavior pattern. Second, the high prevalence of ADHD in JSOs, especially in those with child victims and solo offenders with adolescent/adult victims, might be an indication that impulsivity plays a role in some of the sex offenses. Third, the relation between internalizing problems and sexual offending may have several explanations. On the one hand, internalizing disorders may be the result of previously existing problems with sexuality and/or as a consequence of sexual abuse. JSOs with child victims, for example, frequently reported a history of own sexual victimization, which may have led to internalizing problems other than PTSD. On the other hand, it is assumed that internalizing problems could actually be a reaction to the sexual offenses and its consequences and should not necessarily have preceded the sexual offense [4,32]. If feelings of depression or anxiety have arisen as a result of the sexual offense and/or the police and justice intervention, these conditions may well be a reaction to a shameful, or even threatening, situation. This can be expected to some extend given the aversion from society towards JSOs, and JSOs with child victims in particular. Hence, as no conclusions can be drawn on the causal relationship between disorders and the offenses committed, it may be relevant for treatment purposes to find out whether these internalizing conditions were present before or developed as a consequence of the offense.

As was found in previous research with regard to sexual re-offending (e.g., [21,22]), only a small number of JSOs assessed in the current study was re-referred for another sexual offense (7%). However, as not all sex offenses come to the attention of the police and/or juvenile justice services, the prevalence of re-referrals for another sexual offense could be an underestimation of the actual number of sex offenses.

In addition, a larger group of JSOs was re-referred for a non-sexual offense. Our results are in line with the higher prevalence rate for general re-offending compared to sexual reoffending found in the studies of Caldwell ([21]; sexual reoffending: mean: 7% ± 4%; general reoffending: mean: 43% ± 19%), and Fortune and Lambie [22]. Hence, in some boys the sexual offense seems to be a one-time incident, whereas most boys seem to proceed to general delinquent behavior.

However, our re-referral rate for non-sexual offenses was considerably higher than the re-offense rates found in the aforementioned studies (e.g., [21,22]). At least two reasons might explain this large difference. First, in the current study *reoffending* was defined as a re-referral based on an official judicial registration system, whereas the studies included in the review and meta-analysis of Caldwell [21] and Fortune and Lambie [22] comprised arrest and/or conviction retrieved from official records. The use of convictions might have reduced the mean prevalence rate of general offending in JSOs in these studies. Second, the follow-up period in the current study was in the higher range of the studies included in the Caldwell [21] study and the Fortune and Lambie [22] study. Previous research indicated that the longer the follow-up period, the more likely an offender is to re-offend (e.g., [33]).

With regard to the relationship between mental disorders and reoffending, our results showed that, whereas non-sexual re-referral in JSOs was characterized by the presence of CD, JSOs re-referred for another sexual offense were characterized by the presence of an affective disorder and a history of own sexual abuse. Hence, especially non-sexually reoffending JSOs may be the subgroup of JSOs who commit their sexual offense in the context of a problematic antisocial behavior pattern. In line with expectations, JSOs without any re-referrals had the least mental disorders. With regards to the higher rate of an affective disorder in the group of JSOs referred for another sexual offense compared to those who were not, previous research in general offending juveniles found that affective disorders decreased the risk for reoffending [10]. An explanation for this difference may be that depressive problems in JSOs could be a reaction to the sexual offenses and its consequences as well as the result of previous existing problems with sexual abuse and sexuality.

Findings of this study must be interpreted in the light of some limitations. First, because of the relatively low prevalence of sexual offenses committed by juveniles, it is difficult to include large groups of sex offenders. For that reason, specific subgroups, such as JSOs with child victims (N = 19) or JSOs referred for another sexual offense (N = 7), were small. This also accounts for the prevalence of some mental disorders, such as affective disorders. This may well explain the wide range of the Odds Ratios (ORs), and, therefore, findings need to be replicated in larger samples. Second, at follow up re-referral was determined from an official registration system. Offenses unknown to police and the justice system, the so-called dark number, could, therefore, not be included. In addition, as we examined (re-)referral, not all juveniles had to be convicted for their (sex) offenses. However, acquittal of an offense does not necessarily mean that the (sexual) behavior did not take place. For treatment purposes it is important to examine the prevalence of sexual misbehavior: do JSOs persist in sexual and general delinquent behavior and do they continue to victimize other people? Third, as information regarding mental disorders and the index referrals was cross-sectional, no conclusions can be drawn with regard to causal or time-related paths. Fourth, due to the absence of a general offending control group, it is unknown to what extend the mental disorders are related to sexual deviant behavior or offending behavior in general. Finally, a possible sample bias limits the generalizability of our results. Although responders and non-responders did not differ in age or offense characteristics, responders were more often of non-Dutch ethnicity. As it is suggested that minority and non-minority adolescent offenders differ in prevalence of mental disorders [34], more research on differences in mental disorders between native and non-native JSOs is warranted.

In conclusion, mental disorders are highly prevalent in JSOs, especially in those with child victims. Although most JSOs were re-referred for a non-sexual offense, 7% persisted in their sexual offending behavior. The presence of an affective disorder, a history of sexual abuse, and a lower level of global functioning were more prevalent in JSOs re-referred for another sexual offense than those who did not. The differences in mental disorders between subgroups of JSOs as well as between JSOs re-referred for a sexual offense and JSOs who were re-referred for another (non-sexual) offense are important because of their consequences for treatment as well as for the course of offending behavior. It is clear that this relationship is complex, but of importance for both early intervention and treatment to prevent recidivism, especially sexual recidivism.
